# Early signs of myocardial systolic dysfunction in patients with type 2 diabetes are strongly associated with myocardial microvascular dysfunction independent of myocardial fibrosis: a prospective cohort study

**DOI:** 10.1186/s13098-024-01285-0

**Published:** 2024-02-13

**Authors:** Annemie S. Bojer, Martin H. Sørensen, Stine H. Madsen, David A. Broadbent, Sven Plein, Peter Gæde, Per L. Madsen

**Affiliations:** 1 Department of Cardiology and Endocrinology, Slagelse Hospital, Ingemannsvej 32, 4200 Slagelse, Region Zealand Denmark; 2 Faculty of Health Sciences, Institute of Regional Health Research, University of Southern Denmark, Odense, Denmark; 3 Department of Cardiology, Copenhagen University Hospital Herlev-Gentofte, Herlev, Capital Region of Denmark Denmark; 4 Department of Medical Physics and Engineering, Leeds Teaching Hospitals NHS Trust, Leeds, UK; 5 Leeds Institute of Cardiovascular and Metabolic Medicine, University of Leeds, Leeds, UK; 6 Department of Clinical Medicine, Copenhagen University, Copenhagen, Denmark

**Keywords:** Type 2 diabetes, Cardiovascular complications, Global longitudinal strain, Cardiovascular magnetic resonance imaging, Myocardial microvascular function, Myocardial extracellular volume

## Abstract

**Background:**

Patients with diabetes demonstrate early left ventricular systolic dysfunction. Notably reduced global longitudinal strain (GLS) is related to poor outcomes, the underlying pathophysiology is however still not clearly understood. We hypothesized that pathophysiologic changes with microvascular dysfunction and interstitial fibrosis contribute to reduced strain.

**Methods:**

211 patients with type 2 diabetes and 25 control subjects underwent comprehensive cardiovascular phenotyping by magnetic resonance imaging. Myocardial blood flow (MBF), perfusion reserve (MPR), extracellular volume (ECV), and 3D feature tracking GLS and global circumferential (GCS) and radial strain (GRS) were quantified.

**Results:**

Patients (median age 57 [IQR 50, 67] years, 70% males) had a median diabetes duration of 12 [IQR 6, 18] years. Compared to control subjects GLS, GCS, and GRS were reduced in the total diabetes cohort, and GLS was also reduced in the sub-group of patients without diabetic complications compared to control subjects (controls − 13.9 ± 2.0%, total cohort − 11.6 ± 3.0%; subgroup − 12.3 ± 2.6%, all p < 0.05). Reduced GLS, but not GCS or GRS, was associated with classic diabetes complications of albuminuria (UACR ≥ 30 mg/g) [β (95% CI) 1.09 (0.22–1.96)] and autonomic neuropathy [β (95% CI) 1.43 (0.54–2.31)] but GLS was not associated with retinopathy or peripheral neuropathy. Independently of ECV, a 10% increase in MBF at stress and MPR was associated with higher GLS [multivariable regression adjusted for age, sex, hypertension, smoking, and ECV: MBF stress (β (95% CI) − 0.2 (− 0.3 to − 0.08), MPR (β (95% CI) − 0.5 (− 0.8 to − 0.3), p < 0.001 for both]. A 10% increase in ECV was associated with a decrease in GLS in univariable [β (95% CI) 0.6 (0.2 to 1.1)] and multivariable regression, but this was abolished when adjusted for MPR [multivariable regression adjusted for age, sex, hypertension, smoking, and MPR (β (95% CI) 0.1 (− 0.3 to 0.6)]. On the receiver operating characteristics curve, GLS showed a moderate ability to discriminate a significantly lowered stress MBF (AUC 0.72) and MPR (AUC 0.73).

**Conclusions:**

Myocardial microvascular dysfunction was independent of ECV, a biomarker of myocardial fibrosis, associated with GLS. Further, 3D GLS could be a potential screening tool for myocardial microvascular dysfunction. Future directions should focus on confirming these results in longitudinal and/or interventional studies.

## Background

Diabetic cardiomyopathy is initially characterized by subclinical changes to the myocardial structure. In recent years, global myocardial strain has been demonstrated to detect early signs of myocardial systolic dysfunction in patients with diabetes [1–3]. Strain is a measure of the maximal shortening during cardiac systole in the longitudinal, circumferential, or radial direction as compared to the resting diastolic position. The global longitudinal strain (GLS) of the left ventricle (LV) has been shown to predict future major adverse cardiovascular events in patients with diabetes, particularly the development of heart failure with preserved ejection fraction [4, 5]. Limited knowledge, however, exists on the underlying pathophysiology of decreased strain beyond coronary macrovascular disease.

In recent work, we demonstrated that patients with type 2 diabetes have impaired myocardial perfusion reserve (MPR) [6]. MPR, a quantification of the myocardial microvascular function in the absence of epicardial CAD, was on average 40% lower in patients with diabetes compared to control subjects, which was even further decreased in subjects with other complications of diabetes [6]. Further, we demonstrated that patients with more than ≥ 1 complication of diabetes on average had a 1.6 percentage points higher myocardial extracellular volume (ECV) despite having similar LV mass when compared to control subjects [7]. ECV is an imaging biomarker of interstitial fibrosis [8]. An association of myocardial microvascular dysfunction and extracellular volume with myocardial systolic strain could be the missing link between poor strain and poor cardiovascular outcomes.

Systolic function including global myocardial strain [longitudinal (GLS), circumferential (GCS), and radial (GRS)] as well as myocardial microvascular function and myocardial ECV can be quantified with cardiovascular magnetic resonance (CMR).

We aimed to investigate, in a large population of patients with type 2 diabetes, the hypothesis that imaging biomarkers of coronary microvascular dysfunction and interstitial fibrosis are part of the underlying pathological processes causing early signs of myocardial dysfunction.

## Methods

### Study design and population

Our study protocol has been detailed before [6, 7]. In short, the study was a prospective cross-sectional study of 296 patients with a confirmed diagnosis of type 2 diabetes. The study was registered at www.clinicaltrials.gov (unique identifier NCT02684331). All patients were recruited from the outpatient clinic at Naestved-Slagelse-Ringsted Hospital Department of Endocrinology. The protocol complied with the Declaration of Helsinki and was approved by the local ethics committee of region Zealand (SJ-490) and by the Danish Data Protection Agency (REG-167-2015). The patients were after written informed consent enrolled during the period from 2016 to 2019. We aimed at recruiting a broad population and kept inclusion and exclusion criteria few and simple. Patients were adults between 18 and 80 years of age, with type 2 diabetes for at least 3 months or more. We excluded patients with contraindications to gadolinium-based contrast agents or magnetic resonance imaging or where poor quality (e.g. arrhythmias) would be expected. Thus, we excluded patients with claustrophobia, permanent atrial fibrillation, an estimated glomerular filtration rate (eGFR) < 30 mL/min/1.73 m^2^, or patients with an implanted pacemaker or cardio-defibrillator (contraindication to CMR). Further, for this study, we wanted to assess the effects of microvascular dysfunction not caused by significant macrovascular coronary artery dysfunction. Thus, we excluded patients with confirmed coronary artery disease, with a positive myocardial perfusion imaging test (visually detectable sub-endocardial perfusion defects), or with ischemic late gadolinium enhancement lesions on CMR (sub-endocardial or transmural lesions). Additionally, we included 25 control subjects without diabetes, without cardiopulmonary symptoms, and without known heart disease. In this group, mild hypertension requiring a maximum of one drug treatment as well as statin treatment was allowed. Data on the control population was only used in Table 1 with the purpose of providing the reader with a better understanding of the diabetes population. For all other analyses, only data from patients with diabetes were used.

**Table 1:** Overview of the clinical characteristics and the CMR measures of the total cohort of patients with type 2 diabetes and the patients without any complications to diabetes both compared to the control subjects

	Control subjects	Patients with type 2 diabetes	p-value
n	25	211	
Age	57 [50, 64]	59 [50, 67]	0.3
Sex, %	17 (68)	147 (70)	1.0
Diabetes duration, years	–	12.0 [6.0, 18.0]	
HbA1c, mmol/mol	35 [33, 37]	60 [53, 70]	**
Systolic blood pressure, mmHg	133 (16)	135 (14)	0.3
Diastolic blood pressure, mmHg	81 (10)	82 (9)	0.4
BMI; kg/m^2^	25 (3)	31 (5)	**
Hypertension, no (%)	4 (16)	140 (66)	**
Albuminuria, no (%)	0	Total 73 (35) Mikro 62 (30) Macro 11(5)	
eGFR, mL/min/1.73 m^2^	87 [80, 90]	90 [79, 90]	0.5
Autonomic neuropathy, no (%)	0	66 (32)	
Retinopathy, no (%)	0	57 (27)	
Periphery neuropathy, no (%)	0	80 (40)	
LDL cholesterol (mmol/L)	2.95 [2.18, 3.70]	1.90 [1.40, 2.52]	**
HDL cholesterol (mmol/L)	1.55 [1.20, 1.90]	1.10 [1.00, 1.40]	**
Triglycerides (mmol/L)	1.50 [1.00, 1.80]	2.10 [1.50, 3.20]	**
Cardiac magnetic resonance imaging measures			
LVEDV, mL	166 ± 39	150 ± 32	0.02
LVESV, mL	63 [54, 71]	54 [42, 66]	0.02
LV mass, (g)	122 [105, 136]	133 [110, 157]	0.048
LV mass/BSA, (g/m^2^)	61 ± 8	62 ± 12	0.3
LV mass/LVEDV, g/mL	0.74 ± 0.12	0.91 ± 0.21	**
MPR	5.1 ± 1.5	3.2 ± 1.2	**
MBF rest, mL/min/g	0.63 ± 0.11	0.83 ± 0.19	**
MBF stress, mL/min/g	3.11 ± 0.81	2.56 ± 0.92	0.004
Myocardial ECV, %	27.4 ± 2.1	28.6 ± 3.1	**
LV cardiac output, L/min	6.2 ± 1.3	6.9 ± 1.4	0.01
LV ejection fraction, %	62 ± 4	64 ± 7	0.2
3D global peak strain			
Longitudinal, (%)	− 13.9 ± 2.0	− 11.6 ± 3.0	**
Circumferential, (%)	− 21.6 ± 2.0	− 20.3 ± 3.1	0.041
Radial, (%)	38.5 ± 6.2	34.1 ± 9.4	0.022

*BMI* body mass index, *eGFR* estimated glomerular filtration rate, *LVEDV* left ventricular end-diastolic volume, *LVESV* left ventricular end-systolic volume, *LV* left ventricle, *BSA* body surface area, *MPR* myocardial perfusion ratio, *MBF* myocardial blood flow, *ECV* extracellular volume

**p value < 0.001

### Clinical data

A medical doctor (ASB or MHS) obtained information about prior medical history, and current medication, and performed a physical examination of the patients. Within 14 days CMR imaging was conducted. Blood samples as well as urine samples were obtained during the first encounter with the patients. Albuminuria was defined as a urine albumin-creatinine ratio ≥ 30 mg/g. Retinopathy diagnosis was based on a report from the routine fundoscopy. The autonomic nervous function was assessed with an orthostatic blood pressure assessment and beat-to-beat variability. A beat-to-beat variability of ≤ 6 beats/min and/or a decrease in blood pressure of 25 mmHg or more were used as the diagnostic criteria for autonomic neuropathy. A peripheral neuropathy diagnosis was evaluated from the patient’s last chiropodist report.

### Cardiovascular magnetic resonance imaging

The 1.5 T Siemens Avanto system (Siemens Healthineers, Erlangen, Germany) was used for all patient and control CMR scans. Patients were positioned supine with surface and spine coils, and end-expiratory breath-holds were used during image acquisition. The protocol included cardiac 2-, 3-, and 4-chamber cine images and short-axis steady-state free precession cine images, with a slice thickness of 8 mm and no gap (TE 1.16–1.25 ms; TR 46.24–49.98 ms, matrix 210–208; FoV 258 × 320–485 × 481). Post-processing analyses were performed using CVI42 with semi-automatic contouring (Circle Cardiovascular Imaging, Calgary, Canada, v.5.5.1 or 5.13.5). LV volumes, LVEF, and LV mass were quantified on the short-axis images. Feature tracking 3D global longitudinal, circumferential, and radial strain was quantified automatically in CVI42 using the short axis stack, the 2-, 3-, and the 4-chamber cine sequences [9].

As previously reported [7], ECV was obtained using a MOLLI T1 mapping sequence [18] on basal and mid-ventricular short-axis slices. Native T1 maps (i.e. before gadolinium contrast) were obtained before stress perfusion, while T1 post-contrast maps were acquired ten minutes after contrast injection. The ECV was calculated as:

$$ECV = \left( {1 - haematocrit} \right)\left( {\frac{{{\raise0.7ex\hbox{$1$} \!\mathord{\left/ {\vphantom {1 {T1_{post{\text{-}}contrast, myocardium} }}}\right.\kern-0pt} \!\lower0.7ex\hbox{${T1_{post{\text{-}}contrast, myocardium} }$}} - {\raise0.7ex\hbox{$1$} \!\mathord{\left/ {\vphantom {1 {T1_{native, myocardium} }}}\right.\kern-0pt} \!\lower0.7ex\hbox{${T1_{native, myocardium} }$}}}}{{{\raise0.7ex\hbox{$1$} \!\mathord{\left/ {\vphantom {1 {T1_{post{\text{-}}contrast, blood} }}}\right.\kern-0pt} \!\lower0.7ex\hbox{${T1_{post{\text{-}}contrast, blood} }$}} - {\raise0.7ex\hbox{$1$} \!\mathord{\left/ {\vphantom {1 {T1_{native, blood} }}}\right.\kern-0pt} \!\lower0.7ex\hbox{${T1_{native, blood} }$}}}}} \right).$$

Myocardial perfusion at rest and during adenosine stress (140 µg/kg/min) was quantified on a mid-ventricular short-axis slice following the administration of gadolinium contrast (0.075 mmol/kg Gadovist; Bayer AG) as previously described [10]. The imaging was post-processed using an in-house MATLAB 2015b (MathWorks, Natick, MA) code to quantify myocardial blood flow (MBF). Myocardial perfusion reserve (MPR) was calculated as the ratio of MBF at stress to MBF at rest.

### Statistics

T-test or the Mann–Whitney U test were used to assess differences in LVEF, GLS, GCS, GRS, and other clinical parameters between patients with type 2 diabetes and control subjects. We used ANOVA to assess the impact of non-cardiovascular complications of diabetes: albuminuria, autonomic neuropathy, retinopathy, and peripheral neuropathy. In a simple univariable linear model, the crude associations between myocardial systolic function (GLS, GCS, and GRS) and MBF, MPR, and ECV were assessed. These relationships were then additionally investigated in two multivariable regression models. One model included age, sex, hypertension, and smoking, and a second model with MBF and MPR adjusted for ECV and ECV adjusted for MPR. This last element was included to evaluate the effect of MPR on myocardial systolic function independently from ECV and vice versa. The parameters included in the multivariable model(-s) were chosen based on directed acyclic graphs considering which parameters could confound the association of interest [11].

Receiver operating characteristics (ROC) curves were performed to estimate the area under the curve and thus the specificity and the sensitivity of GLS on the underlying finding of significantly altered MBF, MPR, and ECV. In these analyses impaired MPR and impaired MBF at stress were defined as the mean value minus two standard deviations for the healthy control subjects. Abnormally increased ECV was defined as the healthy control subject's mean plus two standard deviations. All analyses were conducted in R studio version 1.2.1093 (R Development Core Team). A two-sided p < 0.05 was considered statistically significant.

## Results

Of a total of 296 patients initially included in our study, 25 patients declined to participate following the initial examinations. Of the remaining 271 patients, 54 either had a confirmed history of acute or stable coronary artery disease, ischemic late gadolinium enhancement, or a positive myocardial perfusion examination on CMR and were thus excluded from the study. Consequently, we had a final sample size of 211 patients for our analysis.

The clinical characteristics and the CMR measures of the study population are shown in Table 1. Control subjects and patients had normal LV ejection fractions without any significant difference. However, GLS and GRS were lower in patients with type 2 diabetes compared to control subjects, both in the total cohort of patients and in the group of patients with uncomplicated diabetes. As compared to controls, GCS was not lowered in patients with uncomplicated type 2 diabetes compared to control subjects. However, GCS was significantly decreased for the entire patient cohort when compared to control subjects.

### Myocardial systolic strain and non-cardiovascular microvascular complications of diabetes

**Fig. 1:**
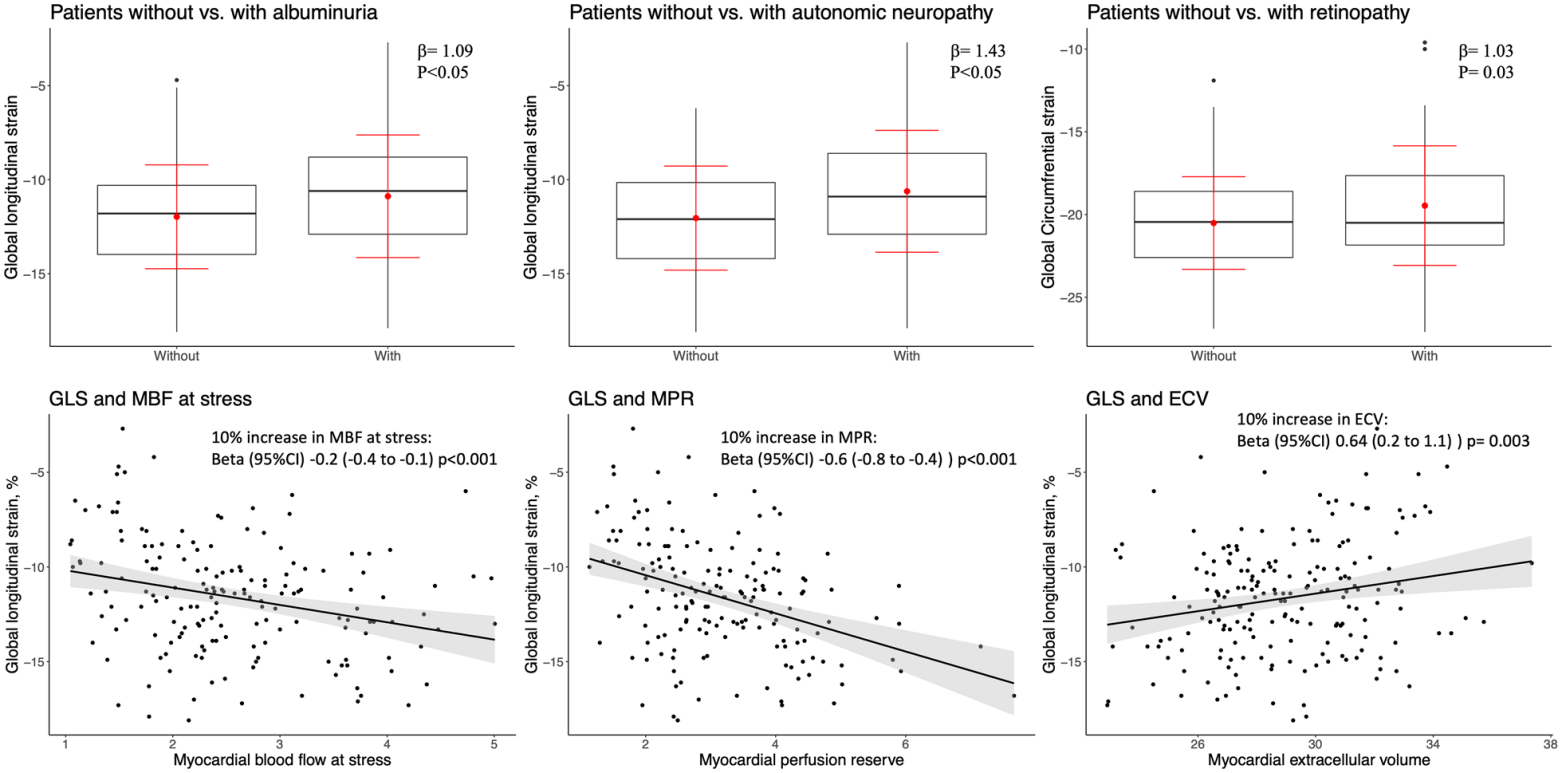
Selected important significant associations with global strain

As shown in Fig. 1, albuminuria was associated with a deterioration in GLS by β = 1.09 percentage points (95% CI 0.22 to 1.96, p < 0.05), and autonomic neuropathy was associated with a deterioration in GLS by β = 1.43 percentage points (95% CI 0.54 to 2.31, p < 0.05). GLS was not associated with retinopathy (β = 0.38 percentage points (95% CI − 0.58 to 1.33), p > 0.05) or peripheral neuropathy [β = 0.72 percentage points (95% CI − 0.17 to 1.61)]. GRS was not associated with any of the four specified complications. Decreasing GCS was associated with retinopathy (β = 1.05 95% CI 0.08 to 2.03), p = 0.03) but not with albuminuria, autonomic neuropathy, or peripheral neuropathy.

### Myocardial systolic deformation, microvascular function, and extracellular volume

In a regression analysis (Table 2), a 10% increase in MBF at rest was associated with improved GCS, the association persisted after adjustment for age, sex, hypertension, and smoking; but after adding ECV to the model the association became non-significant. A 10% increase in MPR and MBF at stress respectively were highly significantly associated with GLS and these associations persisted throughout multivariable adjustments (Table 2 and Fig. 1). In the univariable model (Fig. 1) and in the multivariable model with adjustment for age, sex, hypertension, and smoking, an increase of 10% in ECV was associated with a deterioration of GLS. However, in the model including adjustment for MPR the association between ECV and GLS was no longer significant (Table 2).

**Table 2:** Regression analysis of the association of 10% increased of myocardial microvascular function and myocardial extracellular volume with myocardial systolic global strain parameters

	GLS		GCS		GRS	
	Beta (95% CI)	p	Beta (95% CI)	p	Beta (95% CI)	p
Univariable						
10% MBF rest	0.2 (− 0.0001 to 0.4)	0.05	**− 0.3 (− 0.5 to − 0.1)**	**	**0.6 (0.02 to 1.1)**	0.047
10% MBF stress	**− 0.2 (− 0.4 to − 0.1)**	**	− 0.04 (− 0.2 to 0.07)	0.5	0.1 (− 0.2 to 0.5)	0.5
10% MPR	**− 0.6 (− 0.8 to − 0.4)**	**	0.1 (− 0.07 to 0.4)	0.2	− 0.2 (− 0.9 to 0.5)	0.6
10% ECV	**0.6 (0.2 to 1.1)**	0.003	0.04 (− 0.40 to 0.48)	0.9	− 0.23 (− 1.58 to 1.11)	0.73
Multivariable, age, sex, hypertension and smoking						
10% MBF rest	0.2 (− 0.05 to 0.4)	0.1	**− 0.2 (− 0.4 to − 0.02)**	0.03	0.3 (− 0.3 to 0.9)	0.3
10% MBF stress	**− 0.2 (− 0.4 to − 0.1)**	**	0.05 (− 0.07 to 0.2)	0.4	0.02 (− 0.4 to 0.4)	0.9
10% MPR	**− 0.5 (− 0.8 to − 0.3)**	**	**0.3 (0.02 to 0.5)**	0.035	− 0.2 (− 0.9 to 0.6)	0.7
10% ECV	**0.5 (0.1 to 0.9)**	0.03	0.20 (− 0.24 to 0.64)	0.4	− 0.79 (− 2.20 to 0.61)	0.3
Multivariable where both ECV and MPR are included in the same multivariable model						
10% MBF rest	0.2 (− 0.04 to 0.4)	0.1	− 0.2 (− 0.4 to 0.02)	0.08	0.3 (− 0.3 to 0.9)	0.4
10% MBF stress	**− 0.2 (− 0.3 to − 0.08)**	**	0.08 (− 0.05 to 0.2)	0.2	− 0.01 (− 0.4 to 0.4)	1.0
10% MPR	**− 0.5 (− 0.8 to − 0.3)**	**	**0.3 (0.02 to 0.5)**	0.03	− 0.2 (− 0.9 to 0.6)	0.7
10% ECV	0.1 (− 0.3 to 0.6)	0.5	0.15 (− 0.31 to 0.63)	0.5	− 0.56 (− 2.08 to 0.96)	0.5

*MPR* myocardial perfusion ratio, *MBF* myocardial blood flow, *ECV* extra cellular volume, *GLS* global longitudinal strain, *GCS* global circumferential strain, *GRS* global radial strain. Results with a p value < 0.05 were highlighted in bold

**p value < 0.001

### GLS ability to identify impaired MPR and MBF at stress

**Fig. 2:**
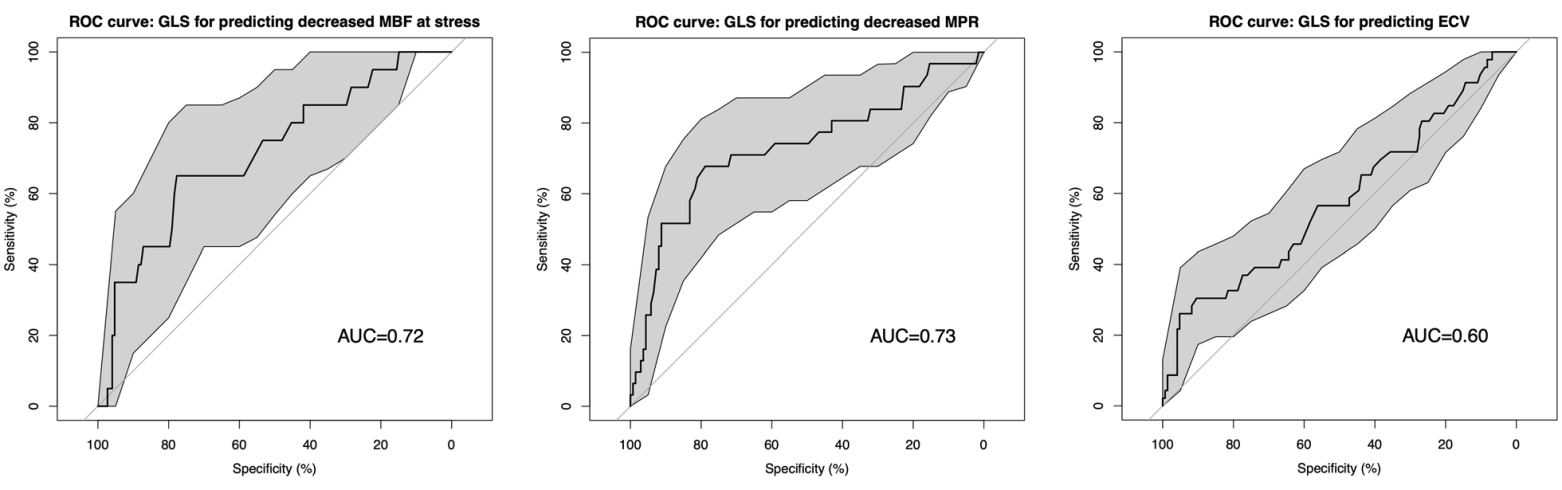
The receiver operating characteristics (ROC) curve showing the diagnostic performance of global longitudinal strain (GLS) to predict myocardial blood flow at stress, myocardial perfusion reserve, and the myocardial extracellular volume

Twenty patients with type 2 diabetes had an MBF at stress as < 1.49 mL/min/g, the threshold for significantly lowered MBF at stress as defined in the methods section. GLS in the two groups was significantly different (normal MBF at stress: − 11.9 ± 2.9 vs. impaired: − 9.62 ± 2.8%, p = 0.001). The receiver operating characteristics (ROC) curve is presented in Fig. 2; the area under the curve (AUC) was 0.72. With a threshold for GLS below − 11.5%, decreased MBF at stress was determined with a sensitivity of 75% and a specificity of 53%. Thirty-one patients with type 2 diabetes had an impaired MPR < 2.1: the GLS in the group of patients with normal *vs*. impaired MPR was − 12.1 ± 2.7 vs. − 9.6 ± 3.3% (p < 0.0001). The ROC curve for GLS to determine impaired MPR is shown in Fig. 2; The AUC was 0.73. With GLS below − 10.8%, significantly lowered MPR could be predicted with a sensitivity of 71% and a specificity of 70%. Forty-six patients had a significantly elevated ECV value above 31.6%. The difference in GLS between ECV groups above and below this threshold was small but statistically significant (− 11.9 ± 2.8 vs. − 10.8 ± 3.4, p = 0.02). Accordingly, the AUC was also small at 0.60 (Fig. 2). With a threshold for GLS at below − 13.1% increased ECV could be predicted with a sensitivity of 72% and a specificity of 34%.

## Discussion

This study uniquely allowed us to evaluate the links between clinically important signs of early deterioration in left ventricular (LV) systolic function and imaging biomarkers of potentially underlying pathophysiology namely: myocardial microvascular function and myocardial interstitial fibrosis. We found that CMR feature-tracking 3D global longitudinal strain (GLS) was significantly reduced (less negative) in patients with type 2 diabetes without ischemic heart disease as compared to healthy control subjects. The deterioration of GLS was associated with albuminuria and autonomic neuropathy, two indicators of poorly controlled diabetes. We demonstrated that a 10% increase in both myocardial perfusion ratio (MPR) and myocardial blood flow (MBF) during stress, hence improved microvascular function, was associated with improved GLS which was independent of myocardial extracellular volume (ECV). ECV was also associated with GLS but not independently, as this association was abolished when MPR was included in a multivariable regression analysis. We also evaluated the capacity of GLS to distinguish between normal and lowered MPR and lowered MBF during stress, and found it demonstrated a moderate level of effectiveness in this regard. Further, GLS only had a poor ability to predict increased ECV.

GLS has been shown to detect early stages of decreased LV function, which is associated with future major adverse cardiovascular events [5]. In patients with type 2 diabetes, Jørgensen et al. found that impaired GLS as determined by echo-Doppler was associated with the development of clinically important cardiovascular disease after a median follow-up of 4.8 years [4]. Certainly, GLS has demonstrated a noteworthy reduction in individuals with heart failure featuring preserved ejection fraction (HFpEF) [12] and has proven valuable in predicting prognosis in heart failure [13]. Despite the effectiveness of Sodium-Glucose co-Transport 2 inhibitors and Glycagone Like Peptide 1 receptor agonists, attempts to address HFpEF beyond these approaches have largely been unsuccessful [14]. To enhance the prospects for managing this condition, a promising avenue involves expanding our comprehension of its pathophysiology. The findings of this study propose that the compromised capacity of myocardial microvascular reserve might, in part, be the missing link between GLS and cardiovascular disease. Consequently, it could serve as a potential focal point for future interventions. Notably, the emphasis here lies on the significance of myocardial microvascular function over interstitial fibrosis as a key focus for attention.

### GLS and non-cardiac microvascular complications of diabetes

Few but robust previous studies have investigated the association between GLS and albuminuria. Our study confirmed the relationship between albuminuria and GLS in patients with type 2 diabetes, similar to findings by Pararajansingam [3] and Mochizuki et al. [15], but in contrast to Jørgensen et al., we observed this association even though most of our patients only had microalbuminuria. Additionally, we demonstrated an association between autonomic neuropathy and GLS, which has not been widely demonstrated before. Although the evidence linking autonomic neuropathy to GLS is not yet robustly established, the association seems logical, given the impact of autonomic regulation on cardiac contractility.

Microalbuminuria and autonomic neuropathy are associated with microvascular disease, and hence these prior studies together with our results support the hypothesis that GLS reflects microvascular disease and not only significant macrovascular coronary artery disease. Also, this may suggest similar pathophysiologic pathways between microvascular kidney injury causing albuminuria and microvascular myocardial injury. We found no association of GRS with microvascular complications, and although significant, GCS was only weakly associated with retinopathy. Concerning the differences between different strain measures, previous literature also reports GLS to be superior to GCS and GRS when it comes to predicting major adverse events [16, 17].

### Relationship of GLS with myocardial microvascular function

Our study found imaging biomarkers of myocardial microvascular function to be robustly associated with GLS. In recent years, several studies have investigated the association between echocardiographic parameters and cardiac microvascular dysfunction in different patient populations without diabetes. In a study of a small cohort of 59 patients without diabetes, a robust inverse correlation was found between coronary flow reserve and GLS, with a correlation coefficient of r = − 0.82 [18]. Another study conducted on patients with recent acute myocardial infarction also reported a strong correlation coefficient (r = − 0.85) between GLS and cardiac flow reserve [9]. A linear association between GLS and coronary microvascular dysfunction was also reported as significant in patients with severe aortic stenosis [19]. Finally, surprisingly, a large study of 963 women without diabetes, who had coronary flow velocity reserve and GLS measured at rest using echocardiography, did not reveal any association between GLS and coronary microvascular dysfunction (defined as a coronary flow reserve below 2.0) [20].

In summary, these studies suggest that GLS may be a valuable tool for detecting early coronary microvascular dysfunction in different patient populations. Microvascular dysfunction is highly prevalent in patients with type 2 diabetes and our study corroborates that a relationship between GLS and coronary microvascular dysfunction is also evident in the group of patients with diabetes.

### GLS as a diagnostic predictor of critical myocardial microvascular dysfunction

We studied a group of patients with diabetes known to be at a very high risk of microvascular myocardial disease. We defined low myocardial microvascular function by using the lower bound of the 95% CI in our healthy control subjects. Other studies have found that a MPR by CMR of < 2.06–2.19 [21, 22] had high specificity and moderate sensitivity for identifying patients with microvascular disease diagnosed by invasive coronary physiology. The threshold we ended with was MPR < 2,1, and this was comparable to other studies.

What we then found was that GLS had a moderate ability to detect critical low myocardial microvascular function. Thus, our study suggests that CMR feature tracking 3D GLS could be used as a diagnostic tool to identify patients with diabetes at risk of microvascular disease.

### Relationship of GLS with myocardial extracellular volume

In our study, an increased ECV was associated with a deterioration of GLS in the univariable model and at first in the multivariable-adjusted regression model, but when MPR was added to the model the association was no longer significant. The statistical explanation for these results could be one of two: as we have demonstrated in previous studies, there is an inverse linear relationship between ECV and the microvascular function [10]. Thus, the results of this study could be due to some collinear effect, and therefore including MPR in the model masked a true albeit small association between ECV and strain. The other explanation is that there is no true association and that the effect of ECV on GLS was just confounded by MPR. Both explanations are plausible. Most animal studies of diabetes have found myocardial interstitial fibrosis to be associated with cardiac diastolic dysfunction without reporting an association with systolic function. In a recent work from our group, we also demonstrated that interstitial fibrosis as evidenced by an increased ECV is associated with the cardiac diastolic function [23], suggesting that interstitial fibrosis is an important part of the pathophysiology leading to cardiac diastolic dysfunction in patients with type 2 diabetes. In this study, however, interstitial fibrosis mainly affected the late phases of diastole related to LV compliance, as the early phases of diastole—like systole as demonstrated in this study—were more related to impaired myocardial perfusion. This could be related to the fact that systole as early diastole is an ATP-dependent process probably more related to perfusion than diffuse fibrosis. Other studies have reported a significant association of ECV with GLS [24], but as mentioned, our study uniquely includes quantification of both interstitial fibrosis and microvascular function.

### Strengths and limitations

Our cohort is the largest cohort to date of patients with type 2 diabetes that has been prospectively examined with gadolinium contrast CMR. This has allowed us to quantify both the microvascular function and the ECV in the population, thus the robust method used in the study is a major strength. Further, we used 3D feature tracking to measure strain. This explains the lower strain values found in the control group compared to previous studies using 2D strain. However, higher reproducibility has been demonstrated with 3D strain and consequently, we choose to use 3D strain instead of 2D strain [9]. Naturally, this study also has limitations. Firstly, our study is single-center cross-sectional, so we cannot comment on causality. Secondly, we excluded patients with an eGFR < 30 mL/min/1.73 m^2^ due to severe kidney disease being a contraindication for gadolinium contrast. Our results should be viewed accordingly.

## Conclusion

The early left ventricular systolic function parameter of global longitudinal strain (GLS) is highly significantly associated with myocardial microvascular function, whereas myocardial extracellular volume, a biomarker of interstitial fibrosis, is not independently associated with GLS. This study also demonstrated that CMR 3D-feature tracking could be used as a diagnostic tool with a moderate discriminatory ability for detecting myocardial microvascular dysfunction in patients with type 2 diabetes. Thus, these results suggest myocardial microvascular dysfunction could be the missing link between GLS and poor outcomes, which should be the focus of future prospective follow-up studies.

## Data Availability

Data are available from the corresponding author upon reasonable request and approvement from Danish authorities.
